# The relation between electroconvulsive therapy and dopamine

**DOI:** 10.1192/j.eurpsy.2023.2266

**Published:** 2023-07-19

**Authors:** C. A. Ciobanu, L. Ionescu, L.-M. Geafer, C.-P. Niculae, A.-M. Ciobanu

**Affiliations:** 1 Faculty of Medicine, “Titu Maiorescu” University, Bucharest; 2 Faculty of Medicine, University of Medicine and Pharmacy “Carol Davila”; 3 Ist Department, Prof. Dr. Alexandru Obregia Psychiatry Hospital, Bucharest, Romania

## Abstract

**Introduction:**

The use of electroconvulsive therapy (ECT) as a treatment for psychotic disorders is well-documented and effective. Despite the fact that ECT is often used, the precise neurobiological mechanisms supporting its effectiveness are still incompletely understood. Over the pastyears, extensive research on primates, rodents, and humans has begun to clarify the effects of electroconvulsive seizures (ECS) and ECT on neurotransmission systems such the dopaminergic system.

**Objectives:**

The aim of this paper is to search evidence in the literature regarding the effects of ECT on the dopamine system.

**Methods:**

In order to write this article, we searched for information in the most important scientific articles from the Google Scholar and Pubmed databases regarding the effects of ECT on the dopaminergic system.

**Results:**

ECT and electroconvulsive shock are linked to enhanced dopamine release and dopamine receptor modification. Human studies show that ECT activates the dopamine system. In a study by Rudorfer et al., it was discovered that ECT increased the amount of homovanillic acid (HVA), a marker of dopamine turnover, in the cerebrospinal fluid (CRF). One important study indicates that monkeys given a brief clinical course of ECT (six sessions only) exhibit significant changes in dopaminergic presynaptic neurotransmission, with baseline function returning to quadratic (‘inverted U’ shape) by six weeks of the last ECT treatment. According to single-unit electrophysiological methods, repeated electroconvulsive shock to rats causes a subsensitivity of dopamine autoreceptors in the substantia nigra. Since effects identical to those reported with repeated treatment were also detected when a single electroconvulsive shock was followed by an acceptable treatment-free interval, this decreased sensitivity is time-dependent.

**Conclusions:**

The results support the idea that ECT boosts the dopamine system and can be an effective strategy in the management of psychotic disorders.

**Disclosure of Interest:**

None Declared

